# Time-course of inflammation and allergic bronchoconstrictor response in a model of ovalbumin-induced rapid pulmonary inflammation in Brown Norway rats

**DOI:** 10.1186/1476-9255-10-S1-P12

**Published:** 2013-08-14

**Authors:** Sorif Uddin, Sara Hughes, Edith Hessel

**Affiliations:** 1 GlaxoSmithKline, Stevenage, UK

## Background

Most animal models that elicit pulmonary allergic inflammation require a protracted sensitisation period that can last for weeks. We describe here further characterisation of a model developed by Werner-Klein and colleagues [1] that exhibits rapid and long lasting pulmonary eosinophilic infiltration requiring only a short sensitisation period. In addition, this modified rat model also exhibits a bronchoconstrictor response to allergen after a single rechallenge two weeks after the initial challenge period.

## Materials and methods

Male Brown Norway rats, 7-9 weeks old, were sensitised to OVA (200µg) emulsified in aluminium hydroxide (2.66mg) via the intraperitoneal route on days 0, 1 and 2. On days 5 and 6, rats were challenged with aerosolised 1% OVA and the bronchoconstrictor response after challenge on day 6 was measured by whole body plethysmography. Bronchoalveolar lavage levels of granulocytes and lymphocytes were ascertained on days 6, 7, 8, 9, 11 and 15. Some animals were challenged again on day 15 with 1% OVA and the resulting bronchoconstrictor response was measured.

## Results

Significant levels of all cell types were present in the BAL from day 7. Eosinophils and CD4 positive lymphocytes in BAL remained at significantly high levels compared to saline challenged animals throughout the time-course investigated. CD8 positive lymphocytes returned back to saline levels by day 15. OVA-induced bronchoconstriction was apparent only after the rechallenge at day 15 while at day 6 it was indistinguishable from saline challenged animals.

## Conclusions

The robust and rapid onset of the inflammatory response in the rat model presented here after a short sensitisation period allows the model to be used for the lead optimisation of novel pharmaceutical agents that modulate allergic airway inflammation. The speed and robustness of the model would also reduce the cost to the animal and so has a 3R’s impact.
